# Regulators of epithelial mesenchymal transition in pancreatic cancer

**DOI:** 10.3389/fphys.2012.00254

**Published:** 2012-07-10

**Authors:** Shin Hamada, Kennichi Satoh, Atsushi Masamune, Tooru Shimosegawa

**Affiliations:** ^1^Division of Gastroenterology, Tohoku University Graduate School of Medicine, SendaiMiyagi, Japan; ^2^Division of Cancer stem cell, Miyagi Cancer Center Research Institute, NatoriMiyagi, Japan

**Keywords:** EMT, pancreatic cancer, BMP, MSX2, miR-126

## Abstract

Pancreatic cancer is a leading cause of cancer-related death due to its invasive nature. Despite the improvement of diagnostic strategy, early diagnosis of pancreatic cancer is still challenging. Surgical resection is the only curative therapy, while vast majority of patients are not eligible for this therapeutic option. Complex biological processes are involved in the establishment of invasion and metastasis of pancreatic cancer and epithelial-mesenchymal transition (EMT) has been reported to play crucial role. EMT is part of the normal developmental processes which mobilizes epithelial cells and yields mesenchymal phenotype. Deregulation of EMT inducing molecules in pancreatic cancer is reported, such as multiple cytokines, growth factors and downstream transcriptional factors. In addition to these molecules, non-coding RNA including miRNA also contributes to EMT. EMT of cancer cell also correlates with cancer stem cell (CSC) properties such as chemoresistance or tumorigenicity, therefore these upstream regulators of EMT could be attractive therapeutic targets and several candidates are examined for clinical application. This review summarizes recent advances in this field, focusing the regulatory molecules of EMT and their downstream targets. Further understanding and research advances will clarify the cryptic mechanism of cancer metastasis and delineate novel therapeutic targets.

## Introduction

Epithelial-mesenchymal transition (EMT) is a normal cellular function which is indispensable during developmental processes such as gastrulation or neural crest cell migration (Pla et al., 2001; Nakaya and Sheng, 2008). However, pancreatic cancer cells misuse this machinery for their invasion toward surrounding tissue and dissemination into distant organs (Rhim et al., 2012). Since invasion and metastasis are the key step for untreatable disease, numerous approaches have been made to prevent EMT of pancreatic cancer cells. Unfortunately, these efforts have not yet conquered the EMT of pancreatic cancer cells due to the complex, cryptic mechanisms involved in this biological process.

Several cytokines and growth factors are reported to induce EMT in pancreatic cancer cells. These factors are derived from cancer cell itself (autocrine) or stromal cell (paracrine). For example, transforming growth factor β (TGFβ) or its family member bone morphogenetic protein (BMP) is reported to cause cellular morphological changes and altered expression of epithelial markers (Fensterer et al., 2004; Hamada et al., 2007). In addition, treatment of pancreatic cancer cells with vascular endothelial growth factor (VEGF) also promotes EMT in pancreatic cancer cells (Yang et al., 2006). These cytokines and growth factors utilize wide variety of receptors and downstream signaling molecules which synergistically or redundantly contribute to the tumor progression.

As a result of the cumulative gene mutations which amplify oncogenic signal, aberrant activation of several signaling pathways are observed in pancreatic cancer cells. Up to 95% of pancreatic cancer harbors constitutively active mutation of *K-ras* oncogene (Furukawa et al., 2006), which leads to the activation of downstream signals for unlimited cellular proliferation. On the other hand, specific gene deletion results in the defect of tumor suppressive signal such as Smad4 deletion, which is observed in 50% of pancreatic cancer (Maitra and Hruban, 2008). Alteration of these signaling pathways also contributes to the EMT induction in pancreatic cancer cells.

Tumor microenvironment also influences the biological behavior of pancreatic cancer. The characteristic feature of pancreatic cancer tissue is dense stroma surrounding tumor cells which is called desmoplastic reaction (Mahadevan and Von Hoff, 2007). The existence of desmoplastic reaction is reported to contribute to pancreatic cancer progression. Recent research identified a significant role of the tumor stromal cells in pancreatic cancer such as protection from chemotherapeutic agents (Muerkoster et al., 2004) or metastasis-promoting role (Xu et al., 2010). Based on these findings, tumor stromal cells are attracting interest as a novel therapeutic target of pancreatic cancer.

Recent advances in the cancer research field identified an additional regulatory molecule of cellular functions. MicroRNA is a member of non-coding RNA consists of 20–23 nucleotide which targets 3′UTR sequence of mRNA for translational repression and destabilization (Farazi et al., 2011). Several reports indicate that microRNA could orchestrate biological processes by targeting hundreds of target mRNAs, including cancer cell invasion and metastasis (Sureban et al., 2011). There are several microRNAs which contribute to pancreatic cancer cell migration and invasion whose expression levels correlate with patients' prognosis (Ali et al., 2010a,b; Giovannetti et al., 2010).

These lines of evidences partially uncovered the complex machinery which keep invasive growth of pancreatic cancer cells. Dissecting the detailed mechanism of EMT in cancer cells will elucidate novel therapeutic targets against invasion and metastasis. Following sections describe current knowledge and future perspectives in this field.

## EMT-inducing cytokines and growth factors

TGFβ is a characteristic cytokine which possesses distinct effects on cellular morphology and proliferation. TGFβ suppresses cellular proliferation accompanied by the induction of cell cycle regulator *p21/waf1*, whose induction requires intact Smad4 (Grau et al., 1997). On the other hand, TGFβ causes down-regulation of epithelial marker E-cadherin (Bardeesy et al., 2006) whose expression attenuates invasive growth of pancreatic cancer cells (Furuyama et al., 2000). Since TGFβ is enriched in plasma or other extracellular sources (Labelle et al., 2011), this cytokine is considered to be an important inducer of EMT during cancer progression. Similarly, a TGFβ family member BMP4 which phosphorylates different Smad (Smad1, 5 and 8) from TGFβ (Smad2 and 3) also induces EMT in pancreatic cancer cells via the induction of MSX2 (Hamada et al., 2007; Gordon et al., 2009). Interestingly, BMP4 also harbors growth inhibitory properties accompanied by the induction of cell cycle regulator *p21/waf1* (Kleeff et al., 1999; Hamada et al., 2009), suggesting redundant roles of these family members which affect pancreatic cancer progression.

Another growth factor is involved in the EMT induction of pancreatic cancer. VEGF is a potent angiogenic factor which stimulates endothelial cell proliferation and migration (Eilken and Adams, 2010). A characteristic tissue structure of pancreatic cancer, the desmoplastic reaction, hampers efficient blood perfusion which gives rise to increased hypoxia within the tumor. Hypoxic condition stabilizes hypoxia-inducible factor 1α (HIF1α) which promotes the transcription of VEGF mRNA (Dery et al., 2005). VEGF also affects cellular morphology of pancreatic cancer cells featured by the loss of polarity, loose cell to cell contact or decreased expression of the epithelial markers E-cadherin and plakoglobin which is in accordance with EMT induction (Yang et al., 2006).

Combination of multiple cytokines and growth factors depicts synergistic effects in EMT induction. Fibroblast growth factor 2 (FGF-2) contributes to the EMT induction in transformed epithelial cells as a downstream effector of *HOXB7*, a homeodomain protein which is overexpressed in breast cancer (Wu et al., 2006). Combined treatment of epithelial cells using recombinant FGF-2 and TGFβ further enhances the migratory phenotype (Shirakihara et al., 2011). This effect is mediated by the altered expression of FGF receptor subtype by TGFβ which sensitizes cells to FGF stimuli. Contribution of multiple cytokines and growth factors during EMT could overwhelm the effect of single-target therapy and enable redundant promotion of invasive growth.

## EMT and intracellular signaling

As mentioned in the previous section, pancreatic cancer cells are under the influence of various cytokines and growth factors. These extracellular stimuli activate intracellular signaling molecules which contribute to the invasive phenotype of cancer cells. Among these signaling molecules, the role of extracellular signal-regulated kinase (ERK) pathway is well characterized during EMT. Treatment of pancreatic cancer cell line Panc-1 by TGFβ leads to the increased phosphorylation of ERK which is indispensable for the EMT induction by TGFβ (Ellenrieder et al., 2001). Since ERK is activated by its upstream regulator Ras/Raf/MEK pathway (Lopez-Chavez et al., 2009), activating mutation of *Kras* could elevate the baseline activity of ERK pathway for further amplification of TGFβ signal.

Besides the Ras/Raf/MEK/ERK pathway, inflammatory signal-related pathway also plays important role for pancreatic carcinogenesis. Previous report suggested that addition of chronic inflammation by caerulein in genetically engineered mice model expressing pancreas-specific *Kras G12D* significantly enhanced the pancreatic cancer incidence (Guerra et al., 2007). Among those inflammatory signal-related molecules, nuclear factor kappa B (NFκB) plays aggravating role by inducing pro-inflammatory cytokines which sustains chronic inflammation within tumor (Ling et al., 2012). NFκB also promotes EMT in pancreatic cancer cells by inducing mesenchymal marker Vimentin and EMT-related transcriptional factor ZEB1 (Maier et al., 2010).

Aberrant activation of other signaling pathway is reported in pancreatic cancer. Notch is a cell surface receptor which regulates cell fate determination and differentiation during embryonic stage whose activation is also seen in pancreatic cancer (Kimura et al., 2007). Acquisition of gemcitabine-resistant phenotype of pancreatic cancer cells is accompanied by the elevated notch activity, whose silencing by siRNA attenuates the EMT phenotype such as vimentin, ZEB1, Slug, and Snail expression (Wang et al., 2009). Another signaling pathway, sonic hedgehog pathway is also involved in pancreatic carcinogenesis whose original role is an endodermal-mesodermal cross-talk during gut development (van den Brink, 2007). Inhibition of hedgehog pathway by several agents such as epigallocatechin-3-gallate or IPI-269609 abrogated EMT phenotype of pancreatic cancer cells (Feldmann et al., 2008; Tang et al., 2012). Targeting these signaling pathways could be an effective therapy for EMT inhibition, but complex cross-talk between multiple pathways could lead to the resistance against single-agent therapy.

## EMT and tumor microenvironment

The tumor microenvironment itself could exert EMT-promoting effects. Hypoxia is a characteristic feature in pancreatic cancer tissue, and besides the VEGF production, hypoxia alters intracellular signals by up-regulating HIF1α. Twist is a transcriptional factor involved in the EMT of pancreatic cancer cells (Satoh et al., 2008) and HIF1α induces its expression (Sun et al., 2009). This is a direct effect of hypoxia on cancer cells similar to the adaptation to the hypoxic conditions (Pasteur effect).

When considering the cellular component within pancreatic cancer stroma, the pancreatic stellate cells (PSC) should be emphasized as a central regulator of fibrosis. (Masamune and Shimosegawa, 2009; Masamune et al., 2009; Erkan et al., 2012) PSCs contribute to the formation of desmoplastic reaction by producing extracellular matrix proteins such as fibronectin or collagen (Bachem et al., 2005). These matrix proteins yield growth stimulatory effects and EMT promotion (Kanno et al., 2008) on pancreatic cancer cells which could be recognized as preconditioning of cancer cells for metastasis.

Another report suggested that PSCs also contribute to establish metastatic site *in vivo* in collaboration with cancer cells (Xu et al., 2010). The detailed mechanism for this phenomenon remains elusive, but recent researches clarified the part of the picture. Indirect co-culture of PSCs promotes EMT phenotype in pancreatic cancer cells independently of TGFβ (Kikuta et al., 2010). This treatment also enhanced the cancer stem cell (CSC) related genes' expression and spheroid formation, a hallmark of CSC function (Hamada et al., 2012a), suggesting novel regulatory mechanism of PSCs in cancer metastasis. Since PSC itself is a non-transformed cell, inhibition of PSC function might be accomplished without acquiring therapy resistance. Targeting cancer supporting cells could be a promising therapeutic option. For the inhibition of PSC function, several signaling pathways are identified such as peroxisome proliferator-activated receptor-gamma, mitogen-activated protein kinases, and reactive oxygen species which could be modulated pharmaceutically (Masamune and Shimosegawa, 2009).

## EMT-inducing microRNA

Recent advances in cancer research field identified novel regulatory molecule in EMT. MicroRNA is a member of non-coding RNA which targets hundreds of target mRNA, thereby orchestrating cellular functions including EMT. Until now, several microRNAs are reported to be involved in the regulation of pancreatic cancer cell motility and invasion. MiR-21 is highly expressed in pancreatic cancer compared with normal tissue, and introduction of miR-21 precursor results in increased cellular proliferation and invasion accompanied by the induction of matrix metalloproteinase-2 and -9 (Moriyama et al., 2009). Furthermore, miR-21 also contributes to the resistance against gemcitabine and correlates with patient's survival (Giovannetti et al., 2010). Expression levels of microRNAs are measurable in plasma samples, and their clinical application is expected. Serum miR-21 expression level is elevated in patient with pancreatic cancer and correlated with poor survival, which indicates miR-21 could be an efficient biomarker of pancreatic cancer (Ali et al., 2010a,b).

In contrast to the miR-21, EMT-inhibiting microRNA is also identified by comprehensive analysis. By comparing the microRNA expression profiles in invasive ductal adenocarcinoma with intraductal papillary mucinous neoplasm, the miR-126 was identified as a significantly down-regulated microRNA in invasive ductal adenocarcinoma (Hamada et al., 2012b). Database analysis identified that miR-126 potentially targets disintegrin and metalloproteinase domain-containing protein 9 (ADAM9), which is highly expressed in pancreatic cancer (Grutzmann et al., 2004). Expression of miR-126 and ADAM9 were mutually exclusive, and re-expression of miR-126 attenuated pancreatic cancer cell migration and invasion (Hamada et al., 2012b). These findings suggest the multimodal regulation of EMT during pancreatic cancer progression.

## Conclusion

**Figure 1:**
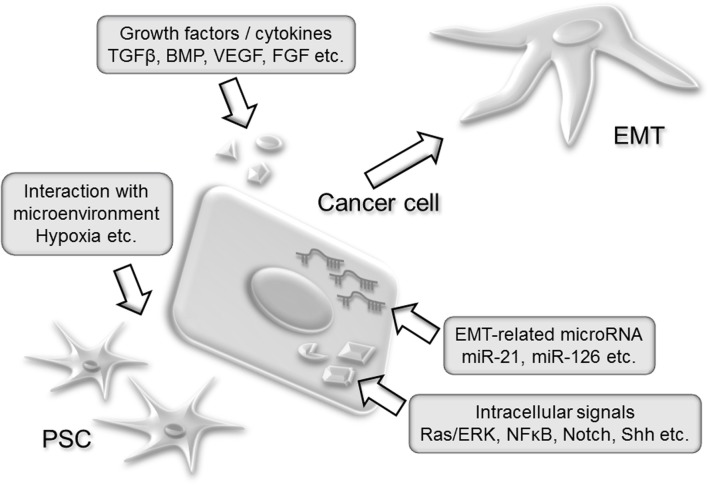
**A schematic view of EMT regulators in pancreatic cancer development.** Secreted cytokines such as TGFβ or BMP activates intracellular signal which leads to the EMT induction. Activating mutation such as *Kras G12D* constitutively stimulates intracellular signal and amplifies extracellular signal. Endogenous alteration of microRNA expression modifies cancer cell function. Stromal cells including PSCs establish protective microenvironment for cancer cells such as desmoplasia.

This review summarized the current knowledge about the regulatory mechanisms of EMT. The schematic view of these regulators of EMT is shown in Figure 1. Inhibition of specific cytokines, growth factors or signaling pathways met their limitations for clinical applications due to the redundant regulation of EMT in pancreatic cancer. Targeting normal cells such as endothelial cells, immune cells, or stromal cells which sustain cancer microenvironment would be novel therapeutic targets against cancer invasion and metastasis. In addition to this concept, a comprehensive regulator of cellular function, microRNA could be a powerful tool in regulating metastasis-promoting microenvironment. Further understanding about the EMT regulation will provide efficient therapy against pancreatic cancer.

### Conflict of interest statement

The authors declare that the research was conducted in the absence of any commercial or financial relationships that could be construed as a potential conflict of interest.
